# Supplementary material for: Stacking Model‐Based Classifiers for Dealing With Multiple Sets of Noisy Labels
Source: Biom J. 2025 Mar 12;67(2):e70042. doi: 10.1002/bimj.70042 (PMC11898607; doi:10.1002/bimj.70042)
Supplement: Supplementary file 1 — Supporting Information [file BIMJ-67-e70042-s001.zip › density-based-ensemble-model-main/competitors_real_data/netlab-master/netlab-master/help/demns1.htm]

Netlab Reference Manual demns1


# demns1

## Purpose

Demonstrate Neuroscale for visualisation.

## Synopsis

```
demns1
```

## Description

This script demonstrates the use of the Neuroscale algorithm for
topographic projection and visualisation. A data sample is generated
from a mixture of two Gaussians in 4d space, and an RBF is trained
with the stress error function to project the data into 2d. The training
data and a test sample are both plotted in this projection.

## See Also

`rbf`, `rbftrain`, `rbfprior`

---

**Pages:**
Index


---

Copyright (c) Ian T Nabney (1996-9)
